# Extraskeletal Intramuscular Chondroma of the Knee – Case Report and Review of the Literature

**DOI:** 10.1055/s-0041-1731796

**Published:** 2021-09-11

**Authors:** Balaji Zacharia, Sanoj Poulose, Govind Sukumara Kurup, Siddarth Mahesh Pawaskar

**Affiliations:** 1 Departamento de Ortopedia, Government Medical College, Kozhikkode, Kerala, Índia; 2 Departamento de Ortopedia, Jubilee Mission Medical College, Trichur, Kerala, Índia

**Keywords:** chondroma, chondromatosis, synovial, knee, soft tissue neoplasms

## Abstract

Extraskeletal chondromas are small nodular cartilaginous lesions not attached to bone or the periosteum. They are rare tumors commonly occurring in the hands and feet. The objective of the present study is to describe a case of extraskeletal intramuscular chondroma (EIC) in the left knee and the diagnostic challenges faced by us.

A 25-year-old female patient presented with slow-growing swelling in the left knee for 2 years. Clinically, the swelling was arising from the quadriceps muscle. We considered possibilities such as rhabdomyoma, neurofibroma, and intramuscular lipoma. Imaging studies suggested a benign fatty tumor. She was treated by excision. Microscopy was consistent with EIC without recurrence.

A rare entity, clinically, EIC can mimic other benign soft-tissue tumors. Histopathology exams can provide a definitive diagnosis. The excision of the tumor is curative.

## Introduction

Soft-tissue chondromas are small well-defined nodular lesions of the cartilage not attached to bone or the periosteum. They are also known as extraskeletal intramuscular chondromas (EICs). First described by Baumuller (1883), 1 the incidence of EIC is of ∼ 1.5% of benign soft-tissue tumors.

They occur in the second to seventh decades of life predominantly in the third and fourth decades. 2 These are slow-growing tumors with a male predominance. The hand and feet are the most common sites. They present as enlarging painful nodules. 3

Rare in the lower extremities, the foot is the most common site for the development of EIC. The thigh, the popliteal region, the knee, and the leg are rare sites. 4 In the present article, we describe a case of EIC in the left knee in a 25-year-old female patient.

## Case Report

A 25-year-old housewife noticed a painless swelling in the inner aspect of the left knee for 2 years. It was slowly increasing in size. There was no fever, constitutional symptoms, or trauma. Her activities of daily living were not affected. There was an oval non-tender swelling (measuring 8 cm × 5 cm × 4 cm) over the anteromedial aspect of the left-knee joint. There were no features of local inflammation. It had smooth surfaces, regular borders, well-defined margins, and firm consistency, arising from the underlying quadriceps muscle. The movements of the knee joint were normal, with no distal neurovascular deficits or local lymph-node enlargement. Rhabdomyoma, neurofibroma, intramuscular lipoma, and bursal swelling were considered as our differential diagnoses.

**Fig. 1:**
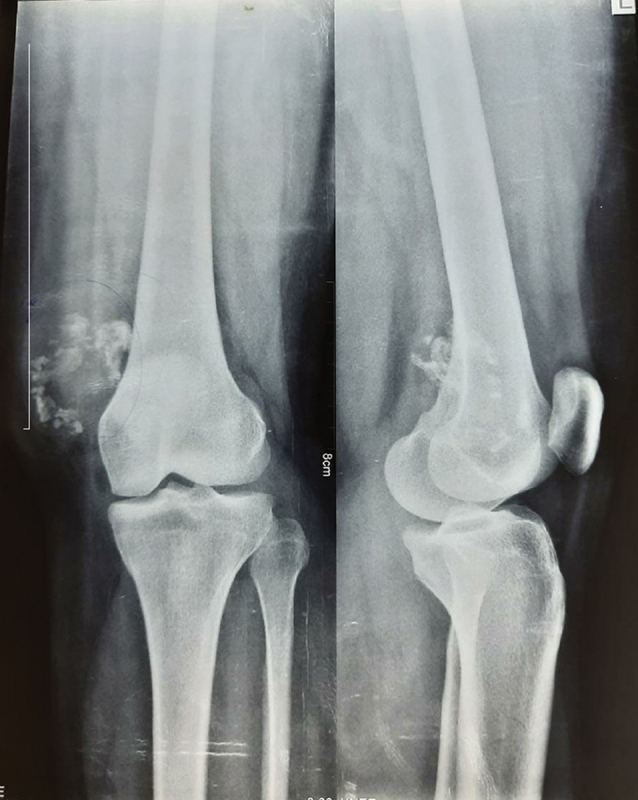
X-ray image of a 25-year-old female patient who presented with slow-growing swelling of the left knee. The radiograph showed a soft-tissue lesion in the anteromedial aspect, with multiple punctuate and circular calcification.

**Fig. 2:**
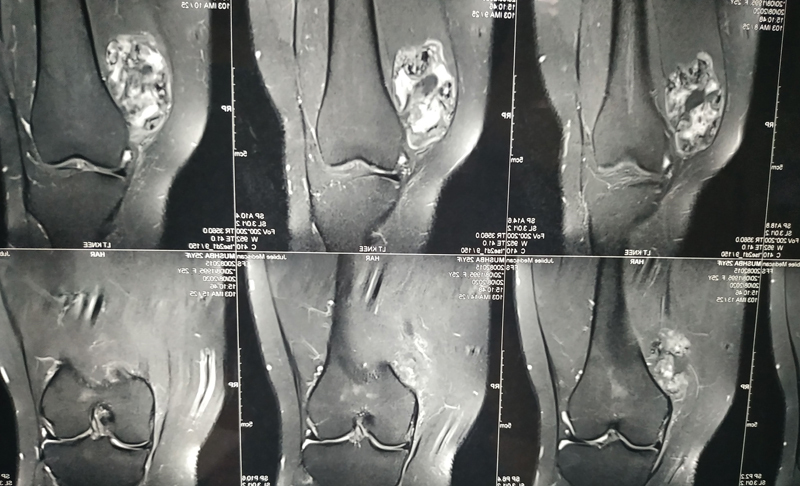
An MRI scan of the patient showing a large soft-tissue mass measuring 6.6 cm × 5 cm × 3.7cm arising from the vastus medialis muscle in the anterior compartment of the left thigh. The lesion had areas with signal intensity similar to that of subcutaneous fat with soft-tissue nodules. There were areas of dystrophic calcification within the lesion. The joint cavity and synovium were unaffected.

Her hemogram, erythrocyte sedimentation rate (ESR), and levels of C-reactive protein were normal. The X-ray image of the left-knee joint showed a soft-tissue lesion in the anteromedial aspect with multiple calcifications ( Fig. 1 ). A magnetic resonance imaging (MRI) scan of the left knee showed a large soft-tissue mass measuring 6.6 cm × 5 cm × 3.7cm arising from the vastus medialis muscle in the anterior compartment of the left thigh. There were areas with signal intensity similar to subcutaneous fat and areas of dystrophic calcification within the lesion. The joint cavity and synovium were unaffected. The adjacent subcutaneous fat and muscle were normal, without surrounding inflammatory changes ( Fig. 2 ). Then, chondroid lipoma, well-differentiated liposarcoma, and malignant fatty tumors became the differential diagnoses.

**Fig. 3:**
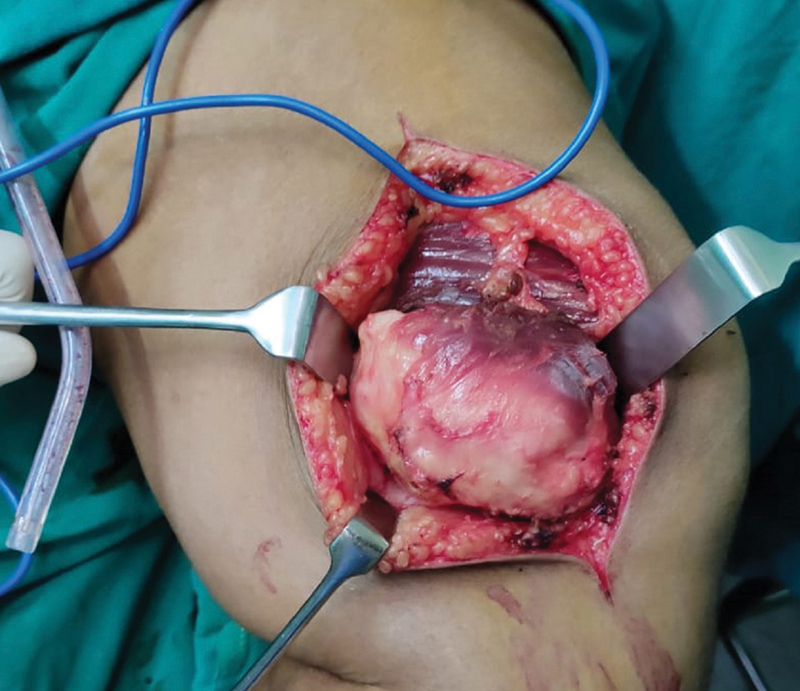
Intraoperative photograph showing a soft-tissue swelling measuring 7 cm × 4 cm × 4 cm arising from the vastus medialis muscle with a pseudocapsule. There was no infiltration into the surrounding soft tissue or bone.

Through a 10-cm long lazy-S incision over the swelling, the skin and subcutaneous tissues were opened, and excision of the swelling, including a cuff of normal muscle, was performed. The swelling was arising from the vastus medialis muscle with a pseudocapsule ( Fig. 3 ).

**Fig. 4:**
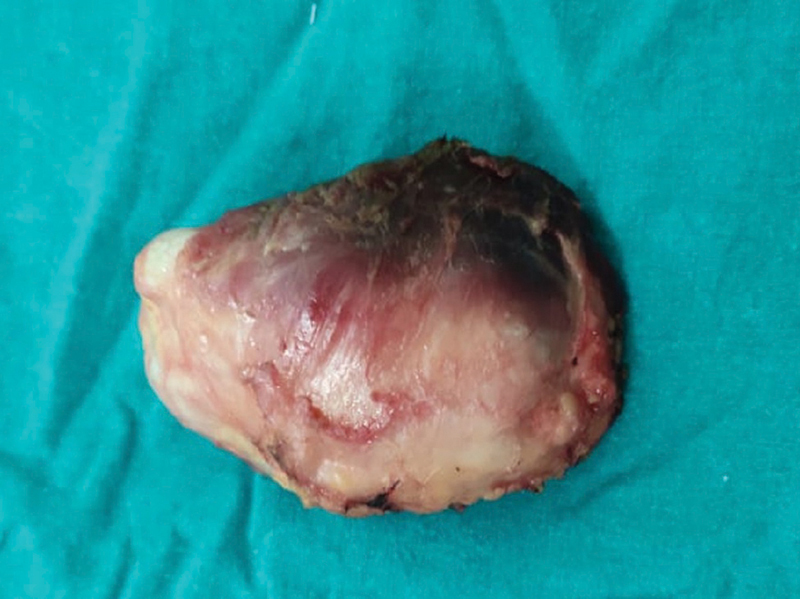
Photograph of the resected specimen.

**Fig. 5:**
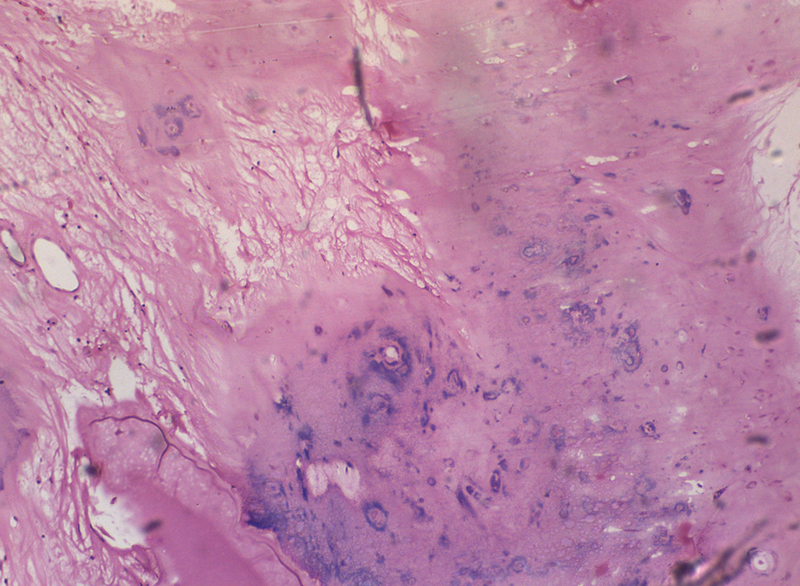
Microscopy of the resected specimen showing nodules of mature hyaline cartilage with lacunar spaces with chondrocytes. Some chondrocytes show lace-like calcification. The cartilaginous tissues are separated by loose fibromyxoid tissue and adipose tissue. Focal areas of ossification were evident. There was no evidence of cellular atypia, mitosis, or necrosis. The resected margins showed normal skeletal muscles.

Macroscopically, the swelling was a well-circumscribed nodular mass measuring 7 cm × 4 cm × 4 cm with attached muscles ( Fig. 4 ). The cut section was greyish-white and yellowish, with gritty and myxoid areas, and the muscles were normal. Microscopically, the tumor was composed of nodules of mature hyaline cartilage with lacunar spaces with chondrocytes. Some chondrocytes show lace-like calcification. The cartilaginous tissues are separated by loose fibromyxoid tissue and adipose tissue. Focal areas of ossification were evident. There was no evidence of cellular atypia, mitosis, or necrosis. The resected margins showed normal skeletal muscles ( Fig. 5 ). The histopathological diagnosis was EIC.

The postoperative period was uneventful. The sutures were removed on the tenth day. The patient was asymptomatic thereafter. No recurrence was observed after three years of follow up. We obtained consent to publish the present report.

## Discussion

Extremely rare soft-tissue tumors, EICs arise without direct attachment to bone or the synovium. There is controversy regarding their origin. Some authors consider that they originate from the synovium, while other authors 3 consider them a developmental fault or metaplasia. Malignant transformation is rarely reported. There are reports of male and female predominance, and EICs are observed near the tendons and tendon sheaths. 3

Radiographically, calcification is evident in 33% to 77% of the cases. The usual patterns of calcification include curvilinear, punctuate, and mixed curvilinear and punctuate types. Magnetic resonance imaging scans show uniform hypointensity in T1- and T2-weighted images of homogenous or dystrophic calcification. The radiographic differential diagnoses are synovial chondromatosis, mesenchymoma, loose bodies, periosteal chondroma, myositis ossificans, crystal-deposition diseases, synovial sarcoma, and malignant soft-tissue chondrosarcoma. 5 Histopathologically, they are highly-cellular tumors with mild to moderate nuclear pleomorphism and scattered mitosis. Areas of focal fibrosis, ossification, and myxomatous changes are observed. Osteoclast-like giant cells and epithelioid cells with granuloma-like proliferation are rarely reported. 6 Supernumerary ring chromosomes of varying sizes and numbers were reported in a case of a soft-tissue chondroma. 7

These lesions are common in the hands and feet. Soft-tissue chondromas have been reported in the masseter muscle, tongue, pharynx, larynx, and other parts of the head and neck region. 8 Extraskeletal intramuscular chondroma can cause trigger finger, symptoms similar to plantar fasciitis, and Hoffa disease. 9 There was another report 10 of posterior interosseous nerve palsy due to compression by a soft-tissue chondroma. The excision of the lesion is curative. Recurrence is rarely reported. 9

Our case was that of a 25-year-old female patient with swelling in the anteromedial aspect of the left knee. Clinically, we considered such possibilities as rhabdomyoma, fibroma, neurofibroma, and a bursa. The lesion was within the quadriceps muscle, and the movement of the knee was restricted when contracting the muscle. Its indolent course and lack of many symptoms prompted us to consider the diagnosis of rhabdomyoma or an intramuscular lipoma. Due to the presence of fatty tissue and calcifications on the MRI scan, the radiological diagnosis was benign lipomatous tumor. But, to our surprise, the microscopic diagnosis was extraskeletal soft-tissue chondroma. This case is herein presented to highlight the rarity of this condition and to describe the clinical and investigative challenges regarding the diagnosis.

Extraskeletal intramuscular chondroma is a rare entity that can clinically mimic other benign soft-tissue tumors. Histopathology exams can provide a definitive diagnosis. The excision of the tumor is curative.
