# Supplementary material for: Extraskeletal Intramuscular Chondroma of the Knee – Case Report and Review of the Literature
Source: Rev Bras Ortop (Sao Paulo). 2021 Sep 11;59(Suppl 1):e1–4. doi: 10.1055/s-0041-1731796 (PMC11254428; doi:10.1055/s-0041-1731796)
Supplement: Related texts

# Research article: Condroma intramuscular extraesquelético do joelho – Relato de caso e revisão da literatura

**Keywords:** condroma, condromatose sinovial, joelho, neoplasias de tecidos moles

## Abstract

Os condromas extraesqueléticos são pequenas lesões cartilaginosas nodulares que não estão aderidas ao osso ou ao periósteo. São tumores raros que ocorrem comumente nas mãos e nos pés. Objetivo deste artigo é descrever um caso de condroma intramuscular extraesquelético (CIE) no joelho esquerdo e os desafios diagnósticos que enfrentamos.

Uma paciente de 25 anos apresentou um edema de crescimento lento no joelho esquerdo havia 2 anos. Clinicamente, o edema era proveniente do músculo quadríceps. Foram consideradas possibilidades como rabdomioma, neurofibroma, e lipoma intramuscular. Os estudos de imagem sugeriram um tumor de gordura benigno. A paciente foi tratada com a excisão cirúrgica. A microscopia indicou CIE sem recorrência.

O CIE é uma lesão rara. Clinicamente, pode ter aspecto semelhante ao de outros tumores benignos dos tecidos moles. A histopatologia pode fornecer um diagnóstico definitivo. A excisão cirúrgica do tumor é curativa.

## Introdução

Os condromas de tecidos moles são pequenas lesões nodulares bem definidas da cartilagem que não estão aderidas ao osso ou ao periósteo. Eles também são conhecidos como condromas intramusculares extraesqueléticos (CIEs). Descritos pela primeira vez por Baumuller (1883). 1 sua incidência é de ∼ 1,5% dos tumores benignos de tecidos moles.

Eles ocorrem entrem a segunda e a sétima décadas de vida, com predominância na terceira e quarta décadas. 2 São tumores de crescimento lento com predomínio no sexo masculino. As mãos e os pés são os locais mais acometidos. Eles se manifestam como nódulos dolorosos e crescentes. 3

O CIE é raro na extremidade inferior, e o pé é o sítio mais comum. A coxa, a região poplítea, o joelho e a perna são raramente acometidos. 4 Neste artigo, descrevemos um caso de CIE no joelho esquerdo de uma paciente de 25 anos.

## Relato de Caso

Uma dona de casa de 25 anos observou um edema indolor na face interna do joelho esquerdo havia 2 anos, que aumentava de tamanho lentamente. Ela não apresentava febre, sintomas constitucionais, ou trauma. Suas atividades cotidianas não foram afetadas. Havia um edema oval indolor (que media 8 cm × 5 cm × 4 cm) na face anteromedial da articulação do joelho esquerdo. Não havia características de inflamação local. O inchaço apresentava superfícies lisas, bordas regulares, margens bem definidas, e consistência firme, e tinha como origem o músculo quadríceps subjacente. Os movimentos da articulação do joelho estavam normais, sem déficits neurovasculares distais ou linfonodomegalia local. Foram considerados como diagnósticos diferenciais rabdomioma, neurofibroma, lipoma intramuscular e edema bursal.

O hemograma, a velocidade de hemossedimentação (VHS) e os níveis de proteína C reativa estavam dentro dos valores de referência. A radiografia da articulação do joelho esquerdo evidenciou lesão de tecidos moles na face anteromedial, com múltiplas calcificações ( Fig. 1 ). A ressonância magnética do joelho esquerdo mostrou uma grande massa de tecido mole que media 6,6 cm × 5 cm × 3,7 cm, e tinha como origem o músculo vasto medial no compartimento anterior da coxa esquerda. Havia áreas com intensidade de sinal semelhante à da gordura subcutânea, e áreas com calcificação distrófica dentro da lesão. A cavidade articular e a sinóvia não foram afetadas. A gordura subcutânea adjacente e o músculo apresentavam aparência normal, sem alterações inflamatórias circundantes ( Fig. 2 ). Os diagnósticos diferenciais foram lipoma condroide, lipossarcoma bem diferenciado, e tumores de gordura malignos.

**Fig. 1:** Radiografia de uma paciente de 25 anos com edema de crescimento lento no joelho esquerdo que evidencia lesão de tecidos moles na face anteromedial com múltiplas calcificações puntiformes e circulares.

**Fig. 2:** A ressonância magnética da paciente mostra uma grande massa nos tecidos moles, medindo 6,6 cm × 5 cm × 3,7 cm, e tendo como origem o músculo vasto medial no compartimento anterior da coxa esquerda. A lesão apresenta áreas com intensidade de sinal semelhante à da gordura subcutânea, com nódulos nos tecidos moles. Havia áreas de calcificação distrófica dentro da lesão. A cavidade articular e a sinóvia não foram afetadas.

Por meio de uma incisão em S aberto ( *lazy S* , em inglês) com 10 cm de comprimento sobre o edema, foram abertos a pele e os tecidos subcutâneos. Foi realizada a excisão do edema, incluindo um manguito do músculo normal. O edema era proveniente do músculo vasto medial, com uma pseudocápsula ( Fig. 3 ).

**Fig. 3:** Imagem intraoperatória mostrando edema nos tecidos moles de 7 cm × 4 cm × 4 cm, tendo como origem o músculo vasto medial, com pseudocápsula. Não houve infiltração nos tecidos moles ou ossos circundantes.

Macroscopicamente, o edema era uma massa nodular bem circunscrita. O tamanho era de 7 cm × 4 cm × 4 cm, com os músculos aderidos ( Fig. 4 ). A seção do corte era branco-acinzentada e amarelada, com áreas de consistência arenosa e mixoide, e os músculos estavam normais. Microscopicamente, o tumor era composto por nódulos de cartilagem hialina madura, com espaços lacunares com condrócitos. Alguns condrócitos apresentavam calcificação em renda. Os tecidos cartilaginosos estavam separados por tecido fibromixoide frouxo e tecido adiposo. As áreas focais de ossificação eram evidentes. Não havia evidência de atipia celular, mitose ou necrose. As margens ressecadas mostravam músculos esqueléticos normais ( Fig. 5 ). O diagnóstico histopatológico foi CIE.

**Fig. 4:** Imagem da amostra ressecada.

**Fig. 5:** Microscopia da amostra ressecada evidenciando nódulos da cartilagem hialina madura, com espaços lacunares com condrócitos. Alguns condrócitos apresentam calcificação em renda. Os tecidos cartilaginosos são separados por tecido fibromixoide frouxo e tecido adiposo. São evidenciadas áreas focais de ossificação. Não havia evidência de atipia celular, mitose ou necrose. As margens ressecadas mostram músculos esqueléticos normais.

O período pós-operatório foi sem intercorrências. As suturas foram retiradas no décimo dia. A paciente ficou assintomática depois disso. Não houve recorrência após 3 anos de acompanhamento. Obtivemos o consentimento para publicar este relatório.

## Discussão

Os CIEs são tumores extremamente raros dos tecidos moles que surgem sem ligação direta ao osso ou à sinóvia. Há controvérsia quanto à origem. Alguns autores consideram que eles se originam da sinóvia, ao passo que outros os consideram uma falha de desenvolvimento ou uma metaplasia. A transformação maligna raramente é relatada. Há relatos de predomínio masculino e feminino, e os CIEs são vistos perto dos tendões e bainhas dos tendões. 3

Radiograficamente, a calcificação é evidente em 33% a 77% dos casos. Os padrões usuais de calcificação incluem os tipos curvilíneos, puntiformes, e os que são um misto de curvilíneo e puntiforme. A ressonância magnética mostra hipointensidade uniforme, com imagens ponderadas em T1 e T2 com áreas de calcificação homogênea ou distrófica. Os diagnósticos diferenciais radiográficos são condromatose sinovial, mesênquimoma, corpos livres, condroma periosteal, miosite ossificante, doenças por depósito de cristais, sarcoma sinovial, e condrossarcoma maligno de tecidos moles. 5 Histopatologicamente, são tumores altamente celulares, com pleomorfismo nuclear leve a moderado e mitose dispersa. São observadas áreas de fibrose focal, ossificação, e alterações mixomatosas. Raramente são relatadas células gigantes semelhantes aos osteoclastos e às células epitelioides, com proliferação semelhante à do granuloma. 6 Foram relatados cromossomos em anel supranumerários de diferentes tamanhos e números em um caso de condroma de tecido mole. 7

Essas lesões são comuns nas mãos e nos pés. Os condromas de tecidos moles foram relatados no músculo masseter, na língua, na faringe, na laringe, e eoutras partes da região da cabeça e do pescoço. 8 O CIE pode causar dedos em gatilho, sintomas semelhantes à fascite plantar, e doença de Hoffa. 9 Houve outro relato de paralisia do nervo interósseo posterior em virtude de compressão por um condroma de tecidos moles. A excisão da lesão é curativa. Raramente é relatada recorrência. 10

Este relato de caso é o de uma paciente de 25 anos com edema na face anteromedial do joelho esquerdo. Clinicamente, foram consideradas possibilidades como rabdomioma, fibroma, neurofibroma, e bursa. A lesão era no músculo quadríceps, e o movimento do joelho se mostrava restringido com a contração do músculo. A evolução lenta e a ausência de muitos sintomas nos levaram a considerar o diagnóstico de rabdomioma ou um lipoma intramuscular. Devido à presença de tecido adiposo e calcificações na ressonância magnética, o diagnóstico radiológico foi de tumor lipomatoso benigno. Porém, para nossa surpresa, o diagnóstico microscópico foi condroma extraesquelético de tecidos moles. Este caso foi apresentado para destacar a raridade desta condição e descrever os desafios clínicos e investigativos enfrentados.

O CIE é uma lesão rara. Clinicamente, pode ser semelhante a outros tumores benignos de tecidos moles. A histopatologia pode fornecer um diagnóstico definitivo. A excisão cirúrgica do tumor é curativa.
